# Synthesis and Characterization of Nanoparticle-Based Dexamethasone-Polypeptide Conjugates as Potential Intravitreal Delivery Systems

**DOI:** 10.3390/ijms24043702

**Published:** 2023-02-12

**Authors:** Natalia Zashikhina, Sergei Gladnev, Vladimir Sharoyko, Viktor Korzhikov-Vlakh, Evgenia Korzhikova-Vlakh, Tatiana Tennikova

**Affiliations:** 1 Institute of Macromolecular Compounds, Russian Academy of Sciences, St. Petersburg 199004, Russia; 2 Institute of Chemistry, Saint-Petersburg State University, St. Petersburg 198504, Russia; 3 Department of General and Bioorganic Chemistry, Pavlov First Saint-Petersburg State Medical University, L’va Tolstogo str. 6-8, St. Petersburg 197022, Russia

**Keywords:** amphiphilic polypeptides, self-assembled nanoparticles, dexamethasone, polymer-drug conjugates, drug delivery systems, intravitreal delivery

## Abstract

The use of dexamethasone for eye disease treatment is limited by its low solubility, bioavailability, and rapid elimination when applied topically. The covalent conjugation of dexamethasone with polymeric carriers is a promising strategy to overcome existing drawbacks. In this work, amphiphilic polypeptides capable of self-assembly into nanoparticles were proposed as potential delivery systems for intravitreal delivery. The nanoparticles were prepared and characterized using poly(L-glutamic acid-co-D-phenylalanine) and poly(L-lysine-co-D/L-phenylalanine) as well as poly(L-lysine-co-D/L-phenylalanine) covered with heparin. The critical association concentration for the polypeptides obtained was in the 4.2–9.4 μg/mL range. The hydrodynamic size of the formed nanoparticles was between 90 and 210 nm, and they had an index of polydispersity between 0.08 and 0.27 and an absolute zeta-potential value between 20 and 45 mV. The ability of nanoparticles to migrate in the vitreous humor was examined using intact porcine vitreous. Conjugation of DEX with polypeptides was performed by additional succinylation of DEX and activation of carboxyl groups introduced to react with primary amines in polypeptides. The structures of all intermediate and final compounds were verified by ^1^H NMR spectroscopy. The amount of conjugated DEX can be varied from 6 to 220 µg/mg of polymer. The hydrodynamic diameter of the nanoparticle-based conjugates was increased to 200–370 nm, depending on the polymer sample and drug loading. The release of DEX from the conjugates due to hydrolysis of the ester bond between DEX and the succinyl moiety was studied both in a buffer medium and a vitreous/buffer mixture (50/50, *v*/*v*). As expected, the release in the vitreous medium was faster. However, the release rate could be controlled in the range of 96–192 h by varying the polymer composition. In addition, several mathematical models were used to assess the release profiles and figure out how DEX is released.

## 1. Introduction

Dexamethasone (DEX) is well known efficient anti-inflammatory, anti-rheumatic, and immunosuppressive drug [1]. It is administered intravenously, orally, and topically to treat various diseases, such as arthritis, edema, multiple myeloma, nasal and eye allergies, and acute and chronic posterior segment eye diseases [2,3].

DEX is a glucocorticoid that, due to its lipophilicity, easily crosses biological membranes. However, its low solubility limits its clinical utility in topical formulations, especially in ocular treatment. A solution to this problem is the use of water-soluble derivatives of DEX, such as DEX sodium phosphate and sodium metasulfobenzoate. However, despite the improved solubility, these drugs have low bioavailability due to low corneal permeability, which is accompanied by the need for frequent DEX administration (3–4 times per day). Furthermore, due to various ocular barriers and route limitations, systemic and topical administration have low efficacy in the treatment of the posterior eye segment [4]. One of the most common local routes to deliver drugs to the posterior segment is intravitreal injections and implants. This route is based on injecting the drug into the intraocular tissue and is commonly performed from the least innervated area of the pars plana [5]. Despite the high efficacy of intravitreal administration, the frequency of administration should be minimized to reduce the traumatic effect and psychological discomfort for the patient. With this aim in mind, various systems of prolonged DEX delivery have been proposed.

Both biodegradable and nondegradable polymers have been examined as carriers for ocular drug delivery [4,5]. The most studied polymers reported as DEX delivery systems are aliphatic polyesters. For example, poly(lactic acid-*co*-glycolic acid) (PLGA) microspheres [6], PLGA nanoparticles [7], triblock-copolymer of PLGA and poly(ethylene glycol) (PEG) thermogel [8], and polycaprolactone (PCL) nanofibers [9] are developed for DEX ocular delivery. Furthermore, dexamethasone-containing Ozurdex^®^ intravitreal implants (Allergan TecAlliance, Dublin, Ireland) consisting of a biodegradable copolymer of lactic and glycolic acids (50/50, *w*/*w*) are currently approved for clinical use [10]. Aliphatic polyester-based systems are capable of prolonging DEX release, but the rate of release depends on the design of the delivery system. In particular, the drug release rate depends on the volume of the polymeric matrix that must degrade to release the entrapped/blended drug. At the same time, the implants require the use of an applicator device (for Ozurdex^®^, the needle diameter is 0.7 mm), whose diameter is significantly larger than that required for the injection of dispersed nanoparticles. When micro- and nanoparticles are used, this is accompanied by a less traumatic effect on the eye. Finally, in the case of micro/nanoparticles, the exact amount of dexamethasone needed for each patient can be conveniently dosed by adjusting the concentration of the particles administered.

Besides aliphatic polyesters, some delivery systems based on modified and unmodified lipid nanoparticles [11,12] and inorganic nanoparticles (porous silicon dioxide [13] and zirconia beads [14]) are considered for DEX delivery in ophthalmology. However, all the types of DEX delivery systems mentioned are quite rigid. Soft nanomaterials (liposomes, micelles, polymersomes, nanogels, etc.) appear to be more advantageous in this regard due to their less damaging soft ocular structures. Taking this into account, such soft nanomaterials as liposomes [15], chitosan nanoparticles covered with hyaluronic acid [16], chitosan oligosaccharide-valylvaline-stearic acid-based nanomicelles [17], cyclodextrin-modified poly(aspartic acid) [18], hyaluronic acid-based micelles [19] are widely studied for eye prolonged treatment.

Besides the delivery of physically entrapped DEX, a few publications on the preparation of conjugated formulations also exist. For instance, poly(amidoamine) (PAMAM) dendrimer-DEX [20], anti-E-selectin-DEX [21], chitosan-DEX [22], mPEG-*b*-poly(L-lysine)-DEX [23], cyclic and linear peptoid-DEX [24], polyallylamine-DEX [25], poly(N-(2-hydroxypropyl)methacrylamide)-DEX [26] conjugates, etc., have been synthesized and characterized. The different conjugation techniques used for DEX can be found in the recent review [27].

In this paper, we focused on the development and characterization of polypeptide-based nanoparticles as potential DEX intravitreal delivery systems. Compared to the widely considered rigid PLA-based nanoparticles, self-assembled polypeptide nanoparticles are soft materials that are more suitable for the eye’s soft structure. Moreover, polypeptides are biocompatible and biodegradable polymers whose rate of biodegradation can be adjusted [28,29]. Additionally, the variation of the macromolecule composition allows the introduction of reactive functionality suitable for drug conjugation [30,31]. Summarizing these positive characteristics of polypeptides, one can expect the suitability of polypeptide nanoparticles as intravitreal drug delivery systems. There are some studies on the development and evaluation of polypeptide hydrogels and soft nanoparticles produced from elastin-like polypeptides or gelatin for subconjunctival or corneal drug delivery [32]. Polysaccharide nanogels modified with L-arginine peptides [33] and silk fibroin-based hydrogels [34] have been considered as platforms for intravitreal delivery systems. To the best of our knowledge, the potential of self-assembled soft nanoparticles based on synthetic polypeptides for intravitreal delivery has not been investigated.

In this study, amphiphilic poly(L-glutamic acid-*co*-D-phenylalanine) (P(Glu-*co*-DPhe)) and poly(L-lysine-*co*-L/D-phenylalanine) (P(Lys-co-D/LPhe)) were used to synthesize amphiphilic polypeptide-DEX conjugates. Both kinds of copolymers tend to self-assemble in aqueous media and can form nanoparticles. The obtained nanosystems were carefully characterized in regard to their size, charge, polydispersity index, stability, and cytotoxicity. Since the vitreous is a negatively charged, viscous medium with hyaluronic acid as its main component, it is hard for positively charged nanoparticles to migrate through it. Taking this into account, the easily conjugated DEX polylysine-based nanoparticles were also coated by strong polyanion heparin. Since heparin is a much stronger polyacid than hyaluronic one, which is a major component of the vitreous gel, the replacement of heparin on the surface of polylysine-based nanoparticles is excluded. DEX release in phosphate saline buffer and porcine vitreous was investigated and discussed.

## 2. Results and Discussion

### 2.1. Polymer Carriers and Their Characteristics

It is known that vitreous is the gel-like fluid that fills an eye. The main components of vitreous humor are collagen fibers and glycosaminoglycans such as hyaluronic acid, some heparan sulfate, and chondroitin sulfate [35]. The anionic nature of the vitreous affects the diffusion of the polymer conjugates and nanoparticles in it. It has been demonstrated that negatively and neutrally charged objects can diffuse inside the eye, whereas cationic systems of any size are immobilized in the vitreous humor by electrostatic interactions with negatively charged polysaccharides [35,36,37]. From this point of view, the DEX conjugates based on negatively charged P(Glu-*co*-Phe) nanoparticles seem to be suitable for intravitreal delivery. However, in comparison to P(Lys-*co*-Phe), the modification of P(Glu-*co*-Phe) with DEX requires an additional step that complicates the synthetic route. In turn, the positive charge of P(Lys-*co*-Phe) restricts its intravitreal administration. Recently, we have shown that coating cationic nanoparticles with a strong polyanion, such as heparin, provides stable polyelectrolyte retention on the surface and switches their properties from cationic to anionic [38,39].

The selected polypeptides were synthesized by ring-opening polymerization of N-carboxyanhydrides (NCA) of α-amino acids using a previously published procedure [40]. The characteristics of the used copolymers, namely, copolymer composition, the ratio of hydrophilic and hydrophobic amino acids, degree of polymerization (*DP*), and polymer dispersity (*Ɖ*), are summarized in Table 1. The used polypeptides have a narrow molecular weight distribution and different ratios of hydrophilic and hydrophobic amino acids in the series.

In the aqueous medium, all used polypeptides were able to self-assemble into monomodal and narrowly dispersed nanoparticles. The characteristics of nanoparticles are presented in Table 2. The hydrodynamic diameter (*D_H_*) and polydispersity index (*PDI*) of nanoparticles were determined by dynamic light scattering (DLS). The *D_H_* values were in the range of 90 to 210 nm, while the polydispersity index (*PDI*) for all nanoparticles was not exceeded 0.3. The polylysine-based nanoparticles had positive surface ζ-potential, as expected, whereas the glutamic acid-based nanoparticles were negatively charged. According to Table 2, the most compact nanoparticles were formed by polypeptides containing higher hydrophobic amino acid content, as well as those in which L-Phe was partially or completely replaced with D-Phe. All amphiphilic polypeptides were self-assembled into nanoparticles at sufficiently low critical association concentrations (CAC) (4.2–9.4 μg/mL in water), which means they are quite stable in aqueous media.

The nanoparticles containing Glu or Lys as a hydrophilic acid had negative or positive surface charges, respectively (Table 2). Covering the Lys-based nanoparticles (P[KF]_DL_-2 sample) with heparin provided a charge switch (sample P[KF]_DL_-2/HEP). Moreover, a strong polyelectrolyte interaction contributed to the compaction of the nanoparticles. Such compaction is explained by the compensation of charges between polycation and polyanion, which partially reduces the repulsion of polymer chains. This phenomenon is well known and has been used to compact large polyanions, such as pDNA, with polycations to enhance nucleic acid delivery inside cells [41].

**Figure 1:**
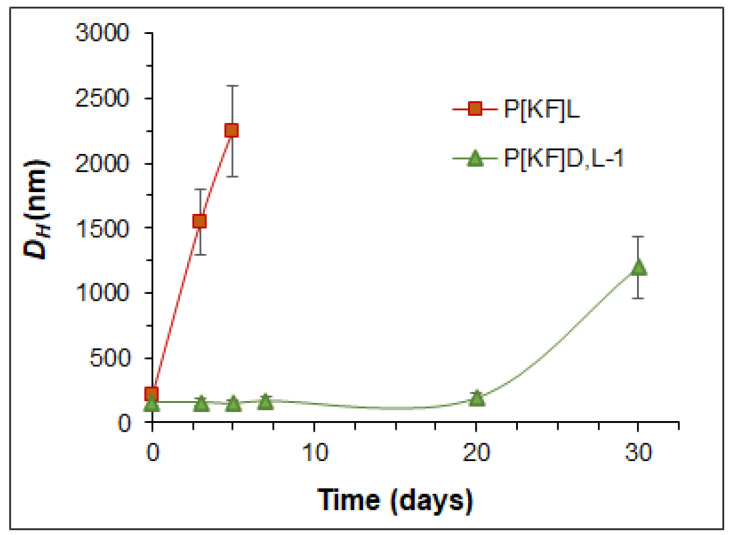
Stability of nanoparticles under simulated physiological conditions (PBS, pH 7.4, containing papain, 37 °C): P[KF]_L_ nanoparticles with initial *D_H_* of 200 ± 20 nm; P[KF]_D,L_ nanoparticles with initial *D_H_* of 110 ± 10 nm.

In addition, the stability of nanoparticles under simulated physiological conditions (0.01 M phosphate saline buffer (PBS) containing papain, 37 °C) was evaluated. The results presented in Figure 1 clearly indicate the low stability of the polypeptide consisting only of L-enantiomers. The P[KF]_L_-based nanoparticles significantly aggregated in the presence of papain within five days due to the detachment of hydrophilic lysine from the nanoparticle surface. The hydrolysis of the surface lysine is followed by the loss of nanoparticles’ stability and aggregation. In turn, the partial replacement of L-Phe (an encoded amine acid) by D-Phe (a noncoded amino acid) contributed to an increase in stability by reducing enzymatic hydrolysis. In this case, the nanoparticles retained their individual characteristics for twenty days. Therefore, the resistance of the polypeptide to degradation can be controlled by varying its composition. 

### 2.2. Mobility of Different Nanoparticles in Vitreous Humor

**Figure 2:**
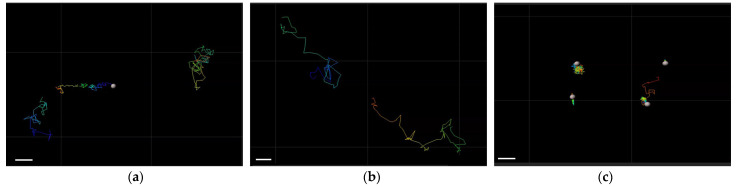
Trajectories of different nanoparticles based in the intact porcine vitreous: anionic P[EF]-2 nanoparticles (**a**), anionic P[KF]_D,L_-2/HEP nanoparticles (**b**)**,** and cationic P[KF]_D,L_-2 nanoparticles (**c**). Nanoparticles based on P[EF]-2 were labeled with Cy5; P[KF]_D,L_-2-based samples were labeled with Cy3 dye. Scale bar corresponds to 2 µm.

The administration of nanoparticles as intravitreal injections requires a study of their diffusion properties in the vitreous gel. The mobility of nanoparticles in the vitreous humor was studied in intact porcine vitreous using cationic P[KF]_D,L_-2 and anionic P[KF]_D,L_-2/HEP and P[EF]-2. The movement of particles in the vitreous was evaluated by the tracking analysis of the fluorescently labeled nanoparticles. After the fluorescently labeled nanoparticles were injected into the vitreous, their trajectory was visualized using confocal fluorescence microscopy. Visualization of the nanoparticles’ trajectory is shown in Figure 2. As seen, the anionic P[KF]_D,L_-2/HEP and P[EF]-2 nanoparticles could migrate in the negatively charged vitreous freely (Figure 2a,b). Importantly, heparin-coated cationic P[KF]_D,L_-2 nanoparticles were stable in the vitreous and behaved like P[EF]-based anionic systems. At the same time, the movement of cationic P[KF]_D,L_-2 nanoparticles was limited by their binding with the vitreous gel (Figure 2c).

The theoretically calculated diffusion coefficients of nanoparticles in water (*D_w_*), and experimentally derived diffusion coefficients of nanoparticles in vitreous gel (*D_v_*) are presented in Table 3. It should be noted that the theoretical diffusion coefficient of nanoparticles in water depends only on their size and differs insignificantly for the selected polymer systems. Diffusion coefficients in the vitreous for anionic nanoparticles decreased by about an order of magnitude as compared to *D_w_*, which is apparently caused by an increase in the viscosity of the system. In turn, the diffusion coefficient for cationic nanoparticles was tens of times lower than for anionic systems. This is due to the electrostatic interactions of cationic nanoparticles with negatively charged polysaccharides in the vitreous humor.

Thus, the vitreous humor is a weak barrier to the diffusion of negatively charged nanoparticles. However, it strongly restricts the mobility of cationic nanoparticles. Consequently, the particle charge is the most important factor regulating the mobility of polymeric particles in the vitreous gel. This result, obtained for the nanoparticles considered in this study, is consistent with the known properties of the mobility of anionic, cationic, and neutral liposomes [35].

### 2.3. Effect of Polypeptide Nanoparticles on ARPE-19 Cell Proliferation

**Figure 3:**
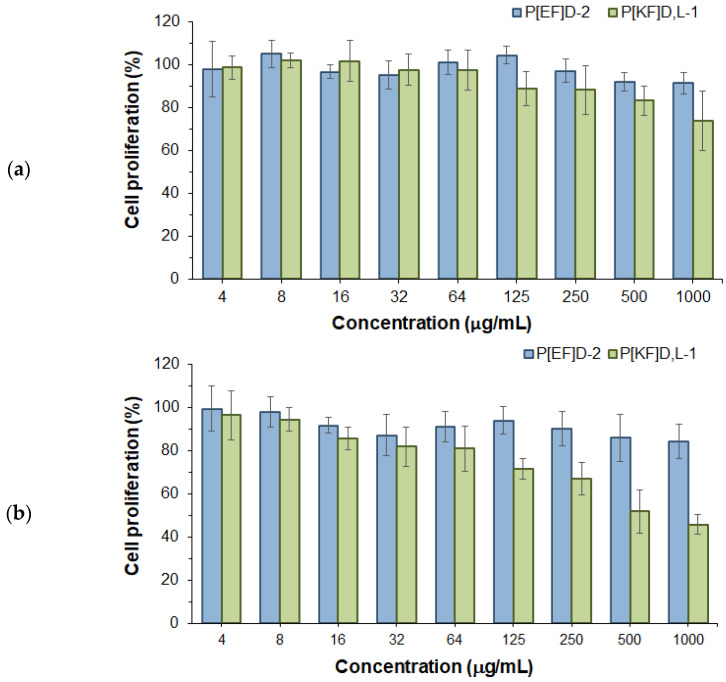
Effect of P[EF]_D_-2 and P[KF]_D,L_-1 nanoparticles on ARPE-19 cell proliferation for 24 h (**a**) and 72 h (**b**).

In order to evaluate the biocompatibility of the nanoparticles under study with retinal cells, the effect of nanoparticles on the proliferation of human retinal pigment epithelial cells (ARPE-19) was studied for 24 and 72 h (Figure 3). Both negatively (P[EF]_D_) and positively (P[KF]_D,L_) charged nanoparticles had no negative effect on ARPE-19 cell proliferation within 24 h. However, incubation of ARPE-19 cells for 72 h in the presence of nanoparticles revealed a difference in cell proliferation. The proliferation of ARPE-19 cells in the presence of high concentrations of P[KF]_D,L_ nanoparticles was significantly reduced compared to control cells. This indicates the toxicity of positively charged nanoparticles for the cells. The obtained result is consistent with previously published findings on the cytotoxicity of the cationic terpolymer based on L-lysine, L-glutamic acid, and L-isoleucine/L-phenylalanine for ARPE-19 cells [42]. At the same time, no decrease in cell proliferation was observed for negatively charged nanoparticles throughout the entire concentration range tested (up to 1000 μg/mL). Thus, the investigated nanomaterials demonstrated high biocompatibility with retinal cells, compared with the widely used polyester nanoparticles. To compare, the PEG-PLGA nanoparticles tested with retinal cells were non-toxic at 200 μg/mL while PLGA nanoparticles coated with hyaluronic acid were non-toxic at 500 μg/mL [43,44].

### 2.4. Synthesis and Characterization of Polypeptide-DEX Conjugates

#### 2.4.1. DEX Modification and General Strategy for Conjugation with Polypeptides

**Figure 4:**
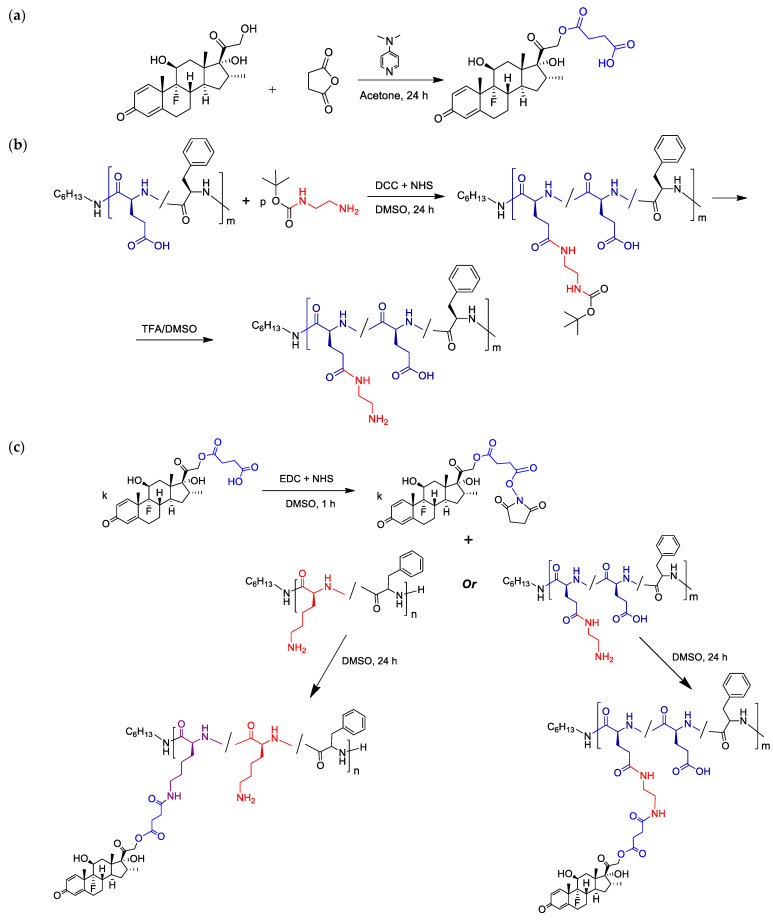
Scheme for the synthesis of DEX-polypeptide conjugates: DEX succinylation (**a**), P[EF] modification with ethylenediamine (**b**), and conjugation of polypeptides with succinylated DEX (**c**).

The general scheme for the synthesis of DEX-polypeptide conjugates is shown in Figure 4. In the first step, the modification of DEX was required to generate the reactive functionality appropriate to conjugation with amino groups of polypeptides. The most developed method for DEX modification is its carboxylation by the succinic anhydrides and their derivatives [22]. DEX has three hydroxyl groups (at the 11th, 17th, and 21th positions), but the OH group at the β-position from the carbonyl group is the most reactive and sterically accessible. In this study, DEX modification was carried out with succinic anhydride in the presence of 4-dimethylaminopyridine (DMAP) (Figure 4a). The structure of the succinylated DEX (DEX-S) was testified by ^1^H NMR spectroscopy (Figure S1, Supplementary Materials). In spectrum of hemisuccinate, the chemical signals of the methylene protons at the β position from the carbonyl group (CH_2_-21) were shifted from 4.08 and 4.50 ppm to 4.81 and 5.06 ppm. In addition, the chemical shift corresponding to the hydroxyl group proton (4.69 ppm) and present in the DEX spectrum (Figure S1a, Supplementary Materials) disappeared in the DEX-hemisuccinate spectrum (Figure S1b, Supplementary Materials), but methylene proton signals appeared at 2.44–2.74 ppm (Figure S1b, Supplementary Materials).

In contrast to lysine-containing polypeptides, which have ε-amino groups, the glutamic acid-based polypeptide has carboxylic groups as the main reactive functionality. To obtain functionality suitable for conjugation with DEX-hemisuccinate, the intermediate modification of P[EF] with BOC-ethylenediamine (BOC-EDA) was performed (Figure 4b). After deprotection, the polypeptide contained free amino groups suitable for further conjugation with activated DEX-S. The conjugation step was identical for P[KF] and modified P[EF] samples (Figure 4c).

#### 2.4.2. Conjugation of DEX with Glutamic Acid- and Lysine-Containing Polypeptides

**Figure 5:**
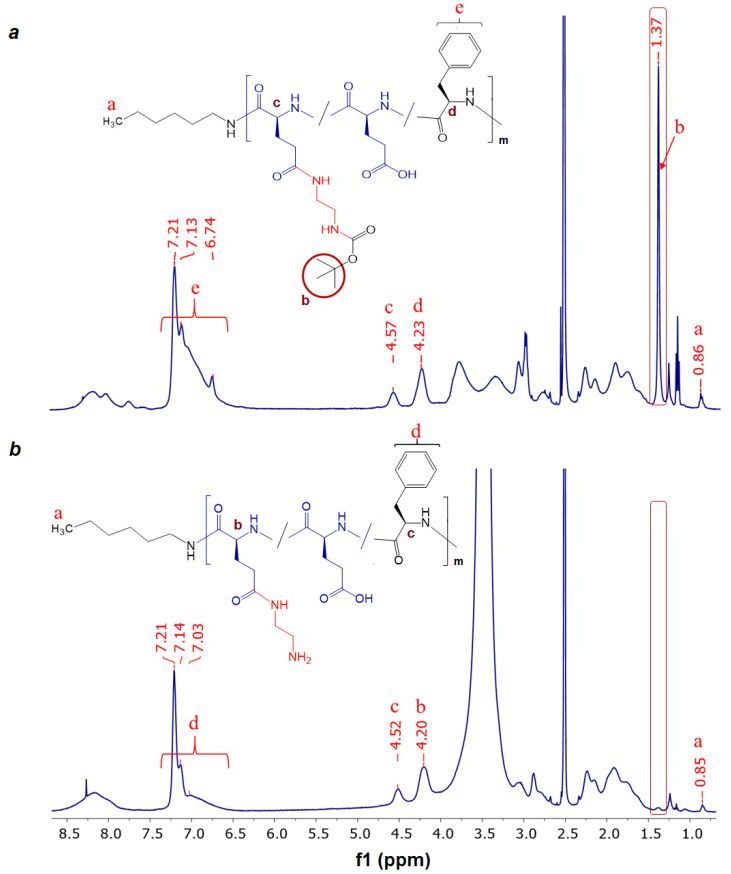
^1^H NMR spectra of P[EF]-EDA(BOC) (**a**) and P[EF]-EDA (**b**) (DMSO-d_6_).

To modify P[EF] with BOC-EDA, 20 mol% of carboxylic groups were activated. The appearance of the Boc group signal in the NMR spectrum of the modified copolymer at 1.38 ppm (Figure 5a) confirmed the success of P[EF] modification with BOC-EDA (Figure 5a). At the same time, this signal is subsequently completely disappearing when the protecting group is removed (Figure 5b). The substitution degree for the P[EF] modification with BOC-EDA was 5.7 mol% from total carboxylic groups in the polymer or 28.5% from the number of activated ester groups. 

**Figure 6:**
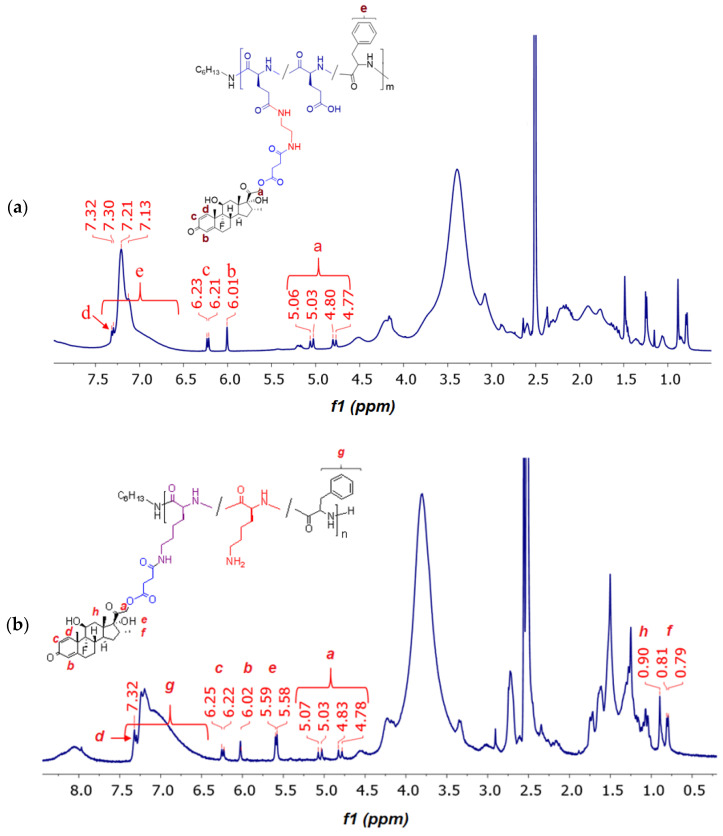
^1^H NMR spectrum of P[EF]-DEX (**a**) and P[KF]-DEX (**b**) conjugates (DMSO-d_6_).

The conjugates of DEX with P[EF]-EDA were prepared by the activation of the DEX-S followed by the reaction of an activated ester with amino groups of polypeptides (Figure 4c). After synthesis, conjugates were purified, freeze-dried, and characterized by ^1^H NMR spectroscopy (Figure 6a). In comparison with the ^1^H NMR spectrum of the P[EF]-EDA copolymer, the chemical shifts typically to DEX (6.01 (s), 6.22 (d), 7.31 (d), 4.70–5.15 ppm) were detected in the spectrum of the conjugate. The presence of DEX signals in the polymer fraction for P[EF]-2-DEX conjugate was also testified by ^1^H NMR DOSY spectroscopy (Figure S2, Supplementary Materials).

The amount of conjugated DEX was determined by qualitative HPLC analysis of the samples obtained after alkaline hydrolysis of ester bonds linking DEX with a succinyl moiety in conjugates. DEX content conjugated to modified P[EF]-2 was 38 μg/mg. The conjugation efficacy was 13%.

The conjugation scheme for P[KF] was similar to that of P[EF]-EDA (Figure 4c). After synthesis, conjugates were purified, freeze-dried, and characterized by ^1^H NMR spectroscopy (Figure 6b). The amount of conjugated DEX was determined, as in the previous case, by qualitative HPLC analysis of samples obtained after alkaline hydrolysis of conjugates.

The effect of the Lys/Phe ratio on the DEX conjugation under equal conditions is shown in Table 4. The conjugation of 42, 52, and 86 μg DEX per mg of polymer for P[KF]_L_, P[KF]_DL_-1 and P[KF]_DL_-2 (corresponds to conjugation efficacy of 21, 26 and 43%), respectively. For both series of polypeptides (P[EF] and P[KF]), the DEX fraction in conjugates increased with the increase in the ratio of hydrophilic/hydrophobic amino acids (Table 4).

**Figure 7:**
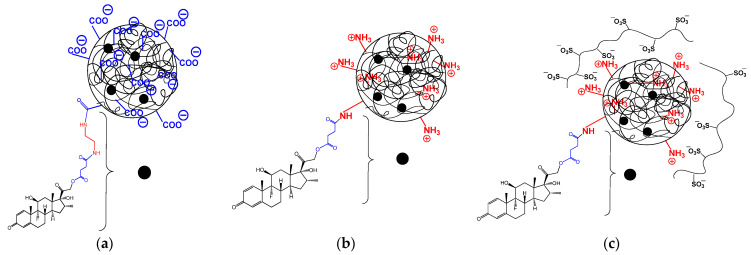
Schematic representation of the different types of self-assembled nanoparticle-based DEX-conjugates: P[EF]-EX (**a**), P[KF]-DEX (**b**) and P[KF]-DEX/HEP (**c**).

All conjugates self-assemble into nanoparticles in aqueous media, forming nanoparticles with hydrodynamic diameters in the range of 200–370 nm, depending on polymer composition. The schematic representation of the different types of nanoparticle-based DEX-conjugates is illustrated in Figure 7**.** As seen, the conjugation of DEX contributed to an increase in the *D_H_* of nanoparticle-based polypeptide nanoparticles (Table 4) in comparison with non-modified nanoparticles (Table 2). Moreover, replacing L-Phe with D,L-Phe in P[KF] samples reduced the hydrodynamic diameter of nanoparticles. This effect is probably attributed to the formation of an ordered secondary polypeptide structure in the case of L-enantiomers and an unordered one in the case of a mixture of enantiomers. Heparin (HEP) coating of P[KF]_DL_ resulted in nanoparticle compaction due to polyelectrolyte interactions and a change in the surface charge from positive to negative.

**Figure 8:**
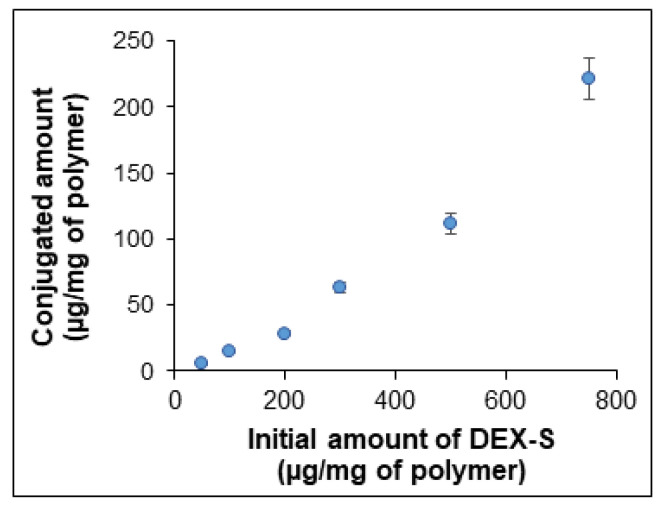
Dependence of DEX-content conjugated to the polypeptide (P[KF]_D,L_-1) on the amount of DEX-S taken for conjugation.

Furthermore, the conjugation efficacy of the P[KF]_D,L_-1 sample was investigated depending on the initial DEX-S amount in the reaction medium. As seen in Figure 8, the content of DEX conjugated to the polymer increased with the growth of the DEX-S amount in the reaction mixture. The conjugated amount varied from 6 ± 1 to 221 ± 10 µg/mg of polymer.

Thus, modification of lysine-based polypeptides with DEX-S is simpler and allows easier variation in DEX loading. At the same time, the positive charge of P[KF] nanoparticles is easily overcome by a simple heparin coating, which provides effective surface recharge. Negative charging the surface of the P[KF] nanoparticles, in turn, completely alters their properties and behavior in the vitreous.

The amount of DEX conjugated with polypeptide nanoparticles is in line with other DEX conjugates. For example, Choksi et al. reported the conjugation of 64 μg of DEX per mg of cationic poly(amidoamine) (PAMAM) dendrimer [20]. Liposomes obtained from this conjugate had a hydrodynamic diameter of 156 nm with a PDI of 0.23. Despite efficient conjugation and appropriate size characteristics, both the PAMAM carrier and its conjugates were found to be quite cytotoxic. Skorik et al. reported the conjugation of 50 and 85 μg of DEX per mg of succinylated chitosan. The DEX-chitosan conjugates formed quite large particles. In particular, depending on the DEX amount, the conjugates revealed hydrodynamic diameters of about 900 nm [22].

**Figure 9:**
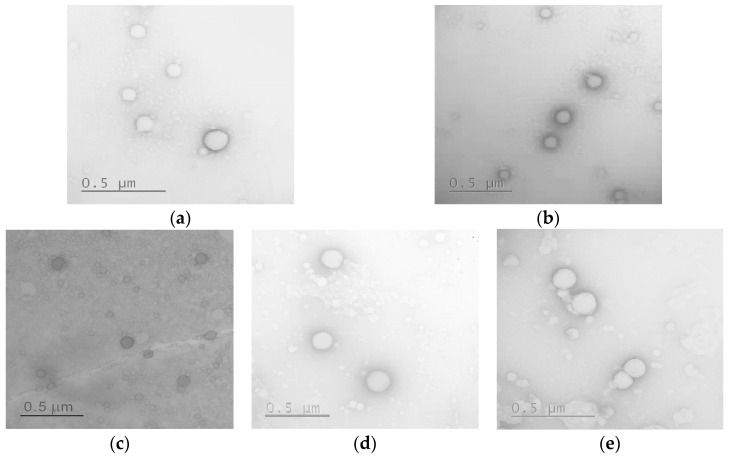
TEM images of nanoparticles and nanoparticle-based polypeptide conjugates with DEX (scale bar 0.5 μm): P[EF]-2 (**a**), P[EF]-2-DEX (**b**), P[KF]_DL_-2 (**c**), P[KF]_DL_-2-DEX (**d**), P[KF]_DL_-2-DEX/HEP (**e**).

The morphology and average diameter of nanoparticles and their conjugates in a dry state were evaluated by transmission electron microscopy (TEM). As seen from the images presented in Figure 9, all nanoparticles were spherical.

The average diameter of nanoparticles calculated from TEM images is summarized in Table 5. It was found that for all the samples studied, the average diameter in the dry state was 20–45% lower than the hydrodynamic diameter. This property is unique to soft nanomaterials and is related to the collapse of solvated or repulsed polymer chains as the sample dries on the grid surface. As a result, the size of the nanoparticles and nanoparticle-based conjugates did not exceed 200 nm.

#### 2.4.3. Storage Stability

**Figure 10:**
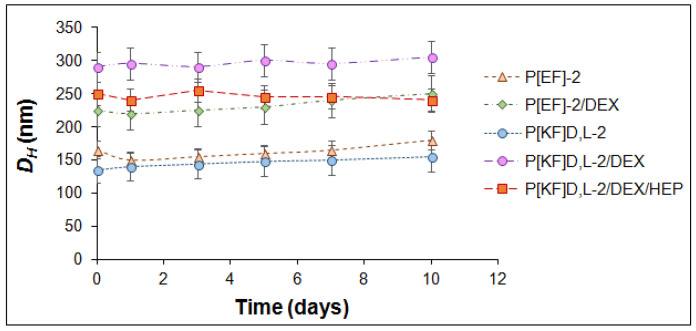
DLS monitoring of hydrodynamic diameters over time for neat nanoparticles and their conjugates with DEX stored at 0.01 M phosphate buffer solution and room temperature (20 °C).

The stability was evaluated by the DLS monitoring of *D_H_* of the nanoparticle-based DEX conjugates for two weeks in 0.01 M phosphate buffer (pH 7.4) at room temperature (20 °C). No changes in the hydrodynamic diameters of nanoformulations were revealed (Figure 10). After two weeks, values of PDI did not exceed 0.2 and 0.3 for the neat nanoparticles and conjugates, respectively (data are not shown). Both neat nanoparticles and their conjugates were stable under these conditions for the period studied.

### 2.5. Release of DEX from Conjugates in Different Media

**Figure 11:**
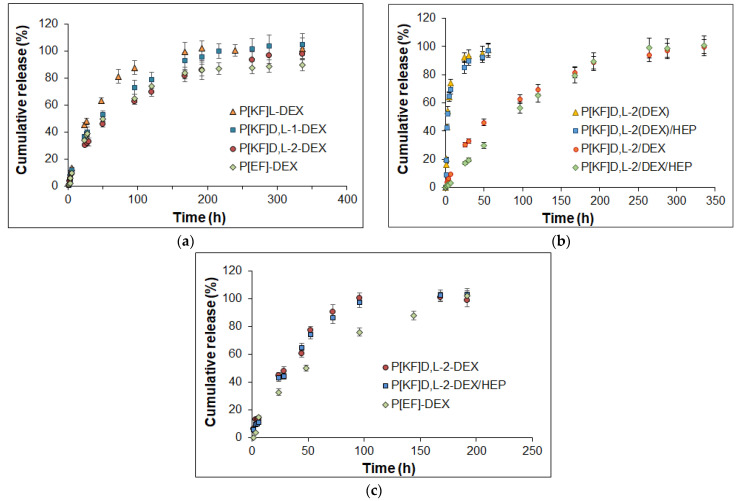
DEX release profiles from the various delivery systems: lysine- and glutamic acid-based conjugates in PBS (**a**), heparin-covered and non-covered lysine-based conjugates (P[KF]_D,L_-2/DEX/HEP and P[KF]_D,L_-2/DEX) and encapsulated nanoparticles (P[KF]_D,L_-2(DEX)/HEP and P[KF]_D,L_-2(DEX)) in 0.01 M PBS (pH 7.4) (**b**), and lysine- and glutamic acid-based conjugates in the vitreous/PBS mixture (50/50, *v*/*v*) (**c**) (incubation at 37 °C).

The release of DEX from the conjugates with polypeptides of different compositions was examined in 0.01 M sodium phosphate buffer containing 0.9% NaCl, pH 7.4 (PBS), and its mixture with porcine vitreous (1/1, *v*/*v*) at 37 °C (Figure 11). The ester bonds formed between DEX and the succinic linker bound to the polypeptide can undergo hydrolysis in aqueous media. After the cleavage of the ester bonds, the subsequent diffusion of DEX from the nanoparticle takes place.

The DEX release profiles were similar for conjugates based on polypeptides of different compositions. The fastest release was observed for P[KF]_L_-DEX, in which total release was achieved within a week. The introduction of D-amino acid into the structure of the polypeptide forming the nanoparticles, as well as an increase in the L-Lys proportion, reduced the rate of DEX release (Figure 11a). The total DEX release from the P[KF]_D,L_-1-DEX and P[KF]_D,L_-2-DEX conjugates were detected after ten and fourteen days, respectively. The slowest release was established for P[EF]-DEX. For this polypeptide, a 90% release was determined after 14 days. P[KF]_D,L_-2-DEX with heparin (P[KF]_D,L_-2-DEX/HEP) reduced the release during the first days, but then the release profile was the same as for its uncovered conjugate precursor (Figure 11b).

Despite the number of published works devoted to the synthesis of DEX-conjugates, only a few studies have addressed the release investigation. Specifically, DEX release in buffer solution within 30 days did not exceed 11% for the chitosan-succinylated DEX conjugates [22]. Although these systems were positioned for intravitreal delivery of DEX, data on cytotoxicity to retinal cells, mobility in the vitreous humor, and release in the vitreous have not been presented by the authors. In addition, the release rate from conjugates of DEX with PEG-*b*-poly(L-lysine) linked with disulfide and ester bonds was studied in buffer media with pH 7.4 and 5.5 [23]. It was found that in both cases in buffer solutions the release did not exceed 10% for five days. In turn, the introduction of glutathione into the buffer with disulfide-bonded DEX was followed by the release acceleration and 70% of DEX was released in five days. Despite the appropriate release kinetics in the buffer media, the systems discussed were developed for anti-inflammatory treatment in cancer therapy. However, they cannot be applied for intravitreal administration owing to their cationic nature.

For comparison with conjugates, the release of DEX encapsulated in the same neat and covered with heparin P[KF]_D,L_-2 nanoparticles was also studied in PBS at 37 °C (Figure 11b). In the case of the encapsulated DEX (P[KF]_D,L_-2(DEX) and P[KF]_D,L_-2(DEX)/HEP), a fast release of the drug was detected within 24 h. In particular, about 80% of the encapsulated DEX was released within the first 8 h, and practical complete release was observed within two days. There was no dramatic difference in the rate of DEX release from the encapsulated P[KF]_D,L_-2(DEX) and P[KF]_D,L_-2(DEX)/HEP systems. However, when the release rates of the conjugated and encapsulated DEX delivery systems based on the same nanoparticles are compared (Figure 11b), the conjugated forms reveal slower drug release.

The DEX release rate for the encapsulated systems observed in this study was comparable to the DEX release rate from other encapsulated soft nanomaterials. In particular, DEX encapsulated in chitosan oligosaccharide nanomicelles [17] or hyaluronic acid-based nanomicelles [19] demonstrated a release of DEX in model buffer media for 4 h and 28 h, respectively. Among the encapsulated polymer systems, the most prolonged release is observed for aliphatic polyester-based solid nanospheres. For example, the release of DEX encapsulated in PLGA nanospheres is completed within twelve days. Increasing the volume of polymer material allows for an increase in drug loading and, as a consequence, prolongs the release. For instance, PLGA microparticles can provide the DEX release within fifty days, while PLGA implants provide the DEX release within about 2–6 months. At the same time, the application of rigid microparticles and implants is more traumatic for the soft tissues of the eye.

The study of the DEX release in the vitreous/PBS mixture (50/50, *v*/*v*) revealed the identical fast release from the P[KF]_D,L_-2-DEX and P[KF]_D,L_-2-DEX/HEP conjugates (Figure 11c). Total release for both conjugates completed within four days. At the same time, the conjugate based on the polypeptide made of L-glutamic acid and D-phenylalanine was slower and took eight days. In general, the rate of DEX release in the biological environment is consistent with the stability of the nanoparticles (Figure 1). Thus, the developed nanoparticle-based polypeptide conjugates with DEX can be considered promising systems for the prolonged intravitreal delivery of dexamethasone.

Mathematical models of in vitro drug release are important for defining the drug release mechanism. In this paper, the obtained DEX release data were fitted with several models (Table 6 and Figure S3 of the Supplementary Materials) to analyze drug release kinetics and mechanisms. First of all, it should be noted, that drug release from all formulations is quite controllable, which follows from high correlation coefficient values (Table 6). One can observe better fitting of release from all formulations to the first-order model than to the zero-order one. Thus, the release of DEX from most formulations under study could be considered dependent on drug concentration according to Fick’s first law. Only P[KF]_D,L_-2/DEX/HEP was nicely fitted with a zero-order model both in PBS and in vitreous, which makes this formulation stand out from the rest ones. Despite the good fit to the first-order model, which indicates the effect of diffusion on the DEX release, the Higuchi model did not show a similar good correlation. It is probably due to the swelling/collapse of the drug-releasing particles. Additionally, the diffusivity of drug molecules could vary within the same formulation due to drug-polymer interactions. For example, this is the case when DEX is present in both non-conjugated and conjugated forms. These factors could also explain the unsatisfactory correlation in the Baker-Lonsdale model.

Korsmeyer-Peppas’ model allowed us to estimate the mechanism by determining the release exponent (*n*, Table 6). Except for the release from P[KF]_D,L_-2/DEX/HEP conjugate, the exponent values were typically between 0.6 and 0.8, indicating anomalous diffusion transport. Both diffusion and polymer relaxation factors affect release in such systems. In the case of P[KF]_D,L_-2/DEX/HEP conjugate the *n* value was close to 1.0, indicating that release occurs via Case II transport mechanisms. It means that release from this formulation is highly affected by the fast increase of water content within the particles as well as by changing the border of swelling. The kinetics of drug-polymer covalent bond destruction are also involved in this process.

Both the Hixson-Crowell and Hopfenberg models, which are based on matrix erosion, fit the release profiles well. The erosion of poly(amino acids) under investigation in an enzyme-free medium could not affect the release within the timeframe of the performed experiment. However, the observed correlation could be explained by a change in particle size not because of erosion but due to the release of DEX, which affects the intermolecular interactions in the particles and hence their density.

Weibull, Gompertz, and Peppas-Sahlin parametric models showed excellent correlation with full release curves for all systems under study and allowed us to estimate some peculiarities of this process. The Weibull model revealed that the release timescale parameter of conjugated DEX (α = 53.228 and 587.182) is greater than that of just encapsulated forms of the drug (α = 2.51 and 2.26). This is obviously due to the covalent attachment of the drug to the polymer within the formulation, which greatly prolongs the release. The curve shape parameter β is different for encapsulated forms, where it is 0.651 and 0.51, and for conjugates, where this parameter was evaluated to be 0.887 and 1.345. When β is less than one, the release curve has a parabolic shape, while when it is greater than one, the curve has a sigmoidal shape. Thus, the addition of HEP to the system changes the form of the release curve.

Gompertz’s model showed that the amount of undissolved drug α is greater in conjugates, while the rate of dissolution β is similar in all cases. A good correlation of release data with this model also indicates the rapid dissolution of the DEX and its immediate release. The Peppas-Sahlin model allowed us to evaluate the contribution of diffusion (K_1_) and relaxation (K_2_) within the release of DEX from formulations under study. Interestingly, in the case of encapsulated formulations and some conjugates (P[KF]D,L-2/DEX and P[EF]-2/DEX), diffusion had a greater effect on release. In the case of the P[KF]_D,L_-2/DEX/HEP conjugate, polymer diffusion and relaxation both contribute to the release process. Thus, it appears that introduction of HEP into the systems under study has the most influence on the mechanism of release.

A comparison of release in PBS and vitreous showed that the rate of release in the second case is a bit faster, but the mechanism of release in both media is generally the same.

## 3. Materials and Methods

### 3.1. Materials

γ-benzyl-L-glutamate (Glu(OBzl)) (≥99%), ε-Z-lysine (Lys(Z), ≥99%), L/D-phenylalanine (L/D-Phe) (≥98%), triphosgene (98%), α-pinene (99%), n-hexylamine (99%), N-hydroxysuccinimide (NHS, 98%), 1-ethyl-3-(3-dimethylaminopropyl)carbodiimide (EDC ≥ 98%), 4-dimethylaminopyridine (DMAP, ≥99%), succinic anhydride (99%), 2-methylanthalic anhydride (98%), *N*,*N*′-dicyclohexylcarbodiimide (DCC, 99%), dexamethasone (≥98%), trifluoromethanesulfonic acid (98%), trifluoroacetic acid (TFA, ≥98%) were purchased from Sigma-Aldrich (Darmstadt, Germany). BOC-ethylenediamine (BOC-EDA, 98%) was a product of Fluorochem (Hadfield, UK). Cy3-NHS (95%) and Cy5-NH_2_ (95%) dyes used for nanoparticle labeling were supplied by Lumiprobe (Moscow, Russia).

Solvents, namely dimethyl sulfoxide (DMSO), dimethylformamide (DMF), dioxane, diethyl ether, ethyl acetate, petroleum ether, ethanol, acetone, as well as triethylamine, were supplied by Vecton (St. Petersburg, Russia) and purified before use. NaH_2_PO_4_ (≥99%) and Na_2_HPO_4_ (≥98%) were purchased from Fluka (Buchs, Switzerland) and used to prepare buffer solutions.

Dialysis membranes with a molecular weight cut-off (MWCO) 3500 (Orange Scientific, Braine-l’Alleud, Belgium) were used for polymer purification. Fresh porcine eyes were received from a slaughterhouse. Intact porcine vitreous samples were prepared exactly as described elsewhere [35].

### 3.2. Methods

#### 3.2.1. Synthesis and Characterization of Delivery Systems

Polypeptides were synthesized by the ROP of NCA. NCAs of Glu(OBzl), Lys(Z) and Phe were synthesized before ROP in freshly distilled and dried solvents using a previously described protocol [45]. Polymerization was carried out in 1,4-dioxane in the presence of *n*-hexylamine as an initiator at 30 °C for 72 h. The molar monomers/initiator ratio was 100. Other details for synthesis and purification can be found elsewhere [38,45]. The spectra were recorded using a Bruker AC-400 NMR spectrometer (400 MHz) (Karlsruhe, Germany). Polymer dispersity was determined for protected polypeptides by the SEC. The analysis was carried out using 0.1 M LiBr in DMF as eluent (0.3 mL/min) at 60 °C. Styragel Column, HMW6E (7.8 mm × 300 mm, 15–20 µm bead size, Waters, Milford, MS, USA) and Shimadzu LC-10 system equipped with refractometric detector (Shimadzu, Kyoto, Japan) were used for analysis. Calculations were made using GPC LC Solutions software (Shimadzu, Kyoto, Japan) and preliminary built calibration curves for poly(methyl methacrylate) standards.

Polypeptide nanoparticles were formed by gradient phase inversion (dialysis) from an organic solvent into deionized water. For this purpose, 5–10 mg of a polypeptide or conjugate was dissolved in 2 mL of DFM and dialyzed against water for 24 h. The samples of formed nanoparticles were freeze-dried and then redispersed under ultrasonication (30 s) in water or 0.01 M PBS (pH 7.4). Hydrodynamic diameter and PDI of the polymer nanoparticles were determined by DLS in phosphate buffer solution (PBS) (C = 0.1–0.2 mg/mL) using a ZetasizerNano-ZS (Malvern, UK) equipped with a He–Ne laser at 633 nm at a scattering angle of 173° and 25 °C. Zeta-potential of the nanoparticles was measured by electrophoretic light scattering (ELS). CAC were measured using conductometry according to a previously published protocol [42].

#### 3.2.2. Stability of Nanoparticles and Movement in Vitreous Humor

The 1.2 mL of the dispersion of nanoparticles in 0.01 M PBS with a concentration of 1 mg/mL, containing 500 µg of papain was incubated within 30 days at 37 °C. The monitoring of the stability of nanoparticles was performed by DLS at predetermined time intervals.

The mobility of labeled nanoparticles in the vitreous humor was recorded with an Andor Neo sCMOS camera mounted on a spinning disk confocal microscope (3i Marianas, Intelligent Imaging Innovations, Denver, CO, USA) equipped with a temperature control system. The analysis was carried out exactly as described elsewhere [35].

The theoretical diffusion coefficient of nanoparticles (*D_w_*) and diffusion coefficient of nanoparticles in the vitreous (*D_v_*) were calculated using the equations below. The theoretical diffusion coefficient in water was calculated using the Stokes-Einstein equation:

(1) $$D_{w}=\frac{k_{b}T}{6\pi \eta r}$$

where *k_b_* is Boltzmann constant (1.38 × 10^−23^ m^2^kg/s^2^K), *T* is a temperature (310.15 K), *η* is the viscosity of water at 37 °C (6.90 × 10^−4^ kg/m·s), *r* is an average radius of nanoparticles obtained from DLS measurements (m).

The diffusion coefficient of nanoparticles in the vitreous was calculated from the slope of the linear part of the mean-square displacement (*MSD*) vs. time plot [35]:

(2) $$D_{v}=\frac{MSD_{\left(\tau \right)}}{2d\tau }$$

where *MSD* was computed with the mobility track analyzer over the entire ensemble of particles, *d* is the dimensionality of the track (*d* = 2 for a 2-demensional track), *τ* is the time delay for the calculated displacement.

#### 3.2.3. Proliferation Assay

Cell proliferation was measured using a 5-bromo-2-deoxyuridine (BrdU; Sigma, USA) incorporation assay. ARPE-19 cells were seeded on the 96-well microplates at a concentration of 1 × 10^3^ cells/well. Cells were cultured for 12 h, and afterward, 20 µL of a 100 µM BrdU solution in DMEM/F12 culture medium was added to each well. The cell DNA was metabolically labeled with BrdU for 24 and 72 h at 37 °C in a humidified atmosphere of a CO_2_-incubator without (control) or with tested polymeric nanoparticles. At the end of the incubation period, cells were fixed in ice-cold 100% methanol at room temperature for 5 min and permeabilized in Triton X-100 permeabilization buffer for 10 min. The incubation of cells with specific antibodies was conducted at room temperature for 1 h. BrdU was detected using fluorescein (FITC) isothiocyanate-conjugated anti-BrdU monoclonal antibody (ThermoFisher Scientific, Waltham, MA, USA). The fluorescence signal was acquired using Varioskan™ LUX multimode microplate reader (ThermoFisher Scientific, Waltham, MA, USA) at FITC excitation and emission spectrum peak wavelengths 495 nm and 519 nm, respectively.

#### 3.2.4. Modification of DEX

Dexamethasone-21-hemisuccinate (DEX-S) was synthesized with slight modifications according to the procedure described elsewhere [21]. For this purpose, 50 mg (0.127 mmol) of DEX, 127 mg of succinic anhydride (1.27 mmol), and 15.9 mg of 4-dimethylaminopyridine (DMAP, 0.13 mmol) were dissolved in 4 mL of acetone. The reaction was carried out under stirring at 22 °C for 24 h. The resulting solution was evaporated to dryness by a rotary evaporator. The reaction product was purified by recrystallization from water/ethanol = 7/3 (*v*/*v*). The white crystals were filtered and dried. The yield of DEX-S was 78%. ^1^H NMR (DMSO-d6), δ (ppm): 0.80 (3H, CH_3_–C16), 0.89 (3H, CH_3_-18), 1.00–1.14 (1H), 1.36 (1H) 1.50 (3H, CH_3_-19), 1.59 (2H), 1.67 (1H), 1.78 (1H), 2.05–2.25 (2H), 2.26–2.46 (2H), 2.55–2.70 (3H, CH_2_—succinic), 2.89 (1H, H-16), 4.16 (1H, H-11), 4.81 (1H, H-21), 5.06 (1H, H-21), 5.18 (1H, OH-17), 5.43 (1H, OH-11), 6.02 (1H, H-4), 6.24 (1H, H-2), 7.30 (1H, H-1), 12.26 (1H, COOH).

#### 3.2.5. Modification of poly(Glutamic Acid-co-Phenylalanine) with Ethylenediamine

Modification of P[EF] copolymers with EDA was carried out with the use of BOC-EDA. In the first step, 827 μL of NHS solution in DMSO (C = 45 mg/mL, 0.4 eq from the amount γ-carboxyl groups of Glu units in copolymer) was added to a solution of 150 mg of P[EF] in 15 mL of DMSO and left for 10 min under stirring. After that, 1.3 mL of a solution of EDC in DMSO (C = 19 mg/mL, 0.2 eq from the amount of γ-carboxyl groups of Glu units in copolymer) was added and stirred for 50 min. In the second step, 1.5 mL of a BOC-EDA solution in DMSO (C = 10 mg/mL, 0.12 eq with respect to the amount of γ-carboxyl groups of Glu units in the copolymer) was added to the mixture obtained at the first step, and the resulting reaction mixture was stirred for 48 h. The modified copolymers were purified by dialysis against DMSO/water (50/50, *v*/*v*) and then water through a membrane with MWCO 3500. After purification, the solution was freeze-dried. The yield P[EF]-EDA-BOC was in the range of 92–98%.

Finally, the protective BOC-group was removed from the linker’s terminal amino group using 50% TFA in DMSO for 2 hours while stirring. After that, TFA was neutralized by TEA (1 eq. to the amount of TFA). After that, the polymer was precipitated with a 4-fold excess of diethyl ether. The dialyzed copolymers were freeze dried and stored at 4 °C before use, as previously described. The yield of P[EF]-EDA was 57–65%.

#### 3.2.6. Synthesis and Characterization of Conjugates

Covalent attachment of dexamethasone to the polypeptide was performed using the activated ester method (Figure 2c). In the first step, a 280 μL NHS solution in DMSO (C = 22.5 mg/mL, 4 eq regarding DEX-S) was added to a 2 mL DEX-S solution in DMSO containing 13.8 mmol DEX-S. The reaction mixture was left to stir for 10 min. After that, a 290 μL EDC/DCC solution in DMSO (C = 14.5 mg/mL, 2 eq regarding DEX-S) was added to the reaction mixture, and the system was left stirring for 1 h. In the second step, the resulting solution was added to a 5 mL solution of the polypeptide from the P[KF] series or P[EF]-EDA in DMSO (C = 5 mg/mL) and left under stirring for 24 h. The unreacted substances were removed by dialysis against DMSO/water (50/50, *v*/*v*) and then water through a membrane with MWCO 3500. The obtained conjugate was freeze-dried and analyzed by ^1^H NMR spectroscopy (DMSO-d6). P[KF]-DEX, δ (ppm): DEX–4.80 (d; H-21), 5.05 (d; H-21), 6.02 (s; H-4), 6.24 (d; H-2), 7.30 (d; H-1), Phe–6.5-7.3 (5H, C_6_H_5_). P[EF]-DEX: DEX–4.79 (d; H-21), 5.05 (d; H-21), 6.01 (s; H-4), 6.22 (d; H-2), 7.32 (d; H-1), Phe–6.6–7.4 (5H, C_6_H_5_).

#### 3.2.7. DEX Release Study

The release of DEX was studied in two media, namely, PBS (pH 7.4) and vitreous/PBS (50/50, *v*/*v*). For this purpose, conjugate dispersions in PBS and vitreous/PBS with a conjugate concentration of 1 mg/mL were incubated at 37 °C for 360 h. At each time point, 40 μL of the reactive medium were taken for HPLC analysis. The release was calculated as a cumulative function. Each sample was analyzed by quantitative HPLC analysis (see below) two times.

Several common mathematical models were applied for the comparison of DEX release from the various conjugated and encapsulated delivery systems. The DDSolver add-in for Microsoft Excel, which is freely available software developed by Zhang Yong and colleagues from China Pharmaceutical University, was used for this purpose [46,47].

#### 3.2.8. DEX Quantitative HPLC Analysis

The amounts of bound and released DEX were quantified by reversed-phase HPLC using the Shimadzu LC-20 Prominence System (Shimadzu, Tokyo, Japan) equipped with a diode-matrix detector and Agilent ZORBAX Eclipse XDB-C18 column (4.6 mm × 150 mm, 5 µm bead size; Santa Clara, CA, USA). The isocratic elution mode was applied using a mixture of acetonitrile and water (30/70, *v*/*v*). DEX elution was detected at 237 nm. The flow rate of the mobile phase was 0.5 mL/min, and the volume of the injection loop was 20 μL. The analysis was performed within 25 min (*t_R_* (DEX) = 21 min). Each sample was analyzed two times. The calibration plot and examples of chromatograms can be found in the Supplementary Materials (Figures S4 and S5).

#### 3.2.9. Morphology and Stability Study

The morphology of nanoparticles was evaluated by TEM. The analysis was carried out with the use of a Jeol JEM-2100 transmission electron microscope (Kyoto, Japan). For TEM analysis, a colloid solution (0.5 mg/mL in water) was dropped at the surface of the 300 mech Cu-grids covered with carbon and formvar. After that, the grid was treated with a 2% (*w*/*v*) uranyl acetate solution for 30–60 s. The excess of the contrasting agent was quickly blotted, and the grid was left for 24 h at room temperature before analysis. The average diameter of the particle was calculated using ImageJ open software developed by the National Institute of Mental Health (Bethesda, MD, USA).

The stability of the hydrodynamic diameter of nanoparticles and nanoparticle-based conjugates at room temperature (20 °C) was monitored by DLS in 0.01 M PBS within 10 days using a ZetasizerNano-ZS (Malvern, UK).

## 4. Conclusions

The obtained results have testified to the applicability of amphiphilic polypeptides containing glutamic acid and lysine for effective conjugation with DEX through a hydrolysable ester bond. In comparison, the use of lysine-containing polypeptides allows us to avoid the additional polymer modification step that is necessary when using a glutamic acid-containing polypeptide. However, the cationic nature of lysine-based polypeptide nanoparticles prevents their mobility in the vitreous humor. This obstacle is overcome by covering lysine-based polypeptide nanoparticles with strong polyanion heparin. Such delivery systems exhibited the same behavior in the vitreous as glutamic acid-based systems, namely, high mobility in the vitreous gel. At the same time, heparin coating had no influence on the DEX release rate in the vitreous gel. The partial replacement of the L-amino acid with D-ones in a polypeptide contributed to the increased stability of nanoparticles in the presence of proteases. In turn, the use of polypeptides as carriers allows for the production of non-toxic and biodegradable polymeric particles, which produce non-toxic amino acids during the biodegradation process. The variation in DEX loading and polymer composition allows for improved dosing of the substance due to its gradual release without the initial explosive release characteristic of encapsulated forms. Based on the results of mathematical modeling, the DEX release occurs according to the diffusion associated with polymer relaxation. The effect of this diffusion proved to be different for the various nanoformulations. In the case of encapsulated DEX, Fickian diffusion predominates. At the same time, DEX conjugation does not significantly change the diffusion rate of the free drug, but the overall release rate changes because of the slow detachment of DEX from the polymer. The release was found to be significantly affected by the coating of the particles with heparin. In this case, the effect of relaxation is as great as that of diffusion. Overall, the proposed nanoparticle-based polypeptide-DEX conjugates seem to be promising as new anti-inflammatory drug formulations for the treatment of eye diseases.

## Figures and Tables

**Table 1:** Characteristics of amphiphilic polypeptides used as carriers for conjugation with DEX.

Copolymer ^a^	Sample Abbreviation	Composition ^b^ (mol%)		[Lys]/[Phe] or [Glu]/[Phe]	*M_n_* (for Protected Copolymers)	*Ð* ^c^
		Lys or Glu	Phe			
P(Glu-*co*-DPhe)	P[EF]-1	74	26	2.8	6700 ^c^	1.19
	P[EF]-2	81	19	4.3	11,220 ^d^	−
P(Lys-*co*-LPhe)	P[KF]_L_	77	23	3.4	12,250 ^c^	1.31
P(Lys-*co*-D,LPhe)	P[KF]_DL_-1	81	19	4.3	11,000 ^c^	1.33
	P[KF]_DL_-2	88	12	7.3	12,150 ^c^	1.37

^a^ Ring-opening polymerization of NCA in 1,4-dioxan using *n*-hexylamine as an initiator; ^b^ For the P[KF] series, the composition was determined from the data of quantitative HPLC analysis of free amino acids obtained after total polymer hydrolysis. For the P[EF] series, the composition was calculated from ^1^H NMR spectra; ^c^ Molecular-weight characteristics and dispersity (*Ɖ*) were determined for Z/OBzl-protected polypeptides using size-exclusion chromatography (SEC) with refractometric detection using poly(methyl methacrylate) standards for column calibration; ^d^ *M_n_* for OBzl-protected polypeptide was calculated from ^1^H NMR spectrum.

**Table 2:** The characteristics of nanoparticles formed due to the self-assembly of amphiphilic polypeptides.

Sample	*D_H_* (nm)	*PDI*	Zeta-Potential ^a^ (mV)	CAC ^b^ (μg/mL)
P[EF]-1	90	0.08	−37 ± 2	n/d
P[EF]-2	165	0.11	−40 ± 2	4.2 ± 0.2
P[KF]_L_	210	0.27	36 ± 1	9.4 ± 0.2
P[KF]_DL_-1	110	0.16	41 ± 1	6.7 ± 0.3
P[KF]_DL_-2	135	0.19	45 ± 2	7.4 ± 0.1
P[KF]_DL_-2/HEP	110	0.22	−20 ± 1	− ^c^

^a^ Measured in water; ^b^ Determined by conductometry in water (n/d—not determined). ^c^ Produced from P[KF]_DL_-2 sample by covering with heparin.

**Table 3:** Mobility of the tested anionic and cationic nanoparticles in the vitreous humor.

Nanoparticles	Surface Charge	*D_w_* (µm^2^/s)	*D_v_* (µm^2^/s)	*D_w_/D_v_*
P[KF]_DL_-2	positive	4.9	0.015 ± 0.06	329
P[KF]_DL_-2/HEP	negative	6.9	0.61 ± 0.10	11.3
P[EF]-2	negative	7.7	0.67 ± 0.15	11.5

**Table 4:** The characteristics of the synthesized nanoparticle-based DEX-polypeptide conjugates.

Nanoparticle-Based Polypeptide-DEX Conjugates	Glu/Phe or Lys/Phe Molar Ratio	DEX Fraction in Polymer (wt%)	*D_H_* (nm)	Zeta-Potential (mV)
P[EF]-1-DEX	2.8	3.0	200 ± 20	−30 ± 3
P[EF]-2-DEX	4.3	3.8	225 ± 35	−35 ± 4
P[KF]_L_-DEX	3.4	4.2	370 ± 30	11 ± 1
P[KF]_DL_-1-DEX	4.3	5.2	320 ± 10	12 ± 1
P[KF]_DL_-2-DEX	7.2	8.6	290 ± 20	15 ± 3
P[KF]_DL_-2-DEX/HEP	7.2	8.6	250 ± 10	−19 ± 1

**Table 5:** Average diameters of nanoparticles and their DEX-conjugated forms are calculated from TEM images.

Sample	Average Diameter Determined by TEM (nm)
P[EF]-2	119 ± 39
P[EF]-2-DEX	181 ± 36
P[KF]_DL_-2	71 ± 20
P[KF]_DL_-2-DEX	178 ± 90
P[KF]_DL_-2-DEX/HEP	158 ± 37

**Table 6:** Correlation coefficients and model parameters were obtained for DEX release profiles of encapsulated and conjugated delivery systems in buffer and vitreous media.

Model	Encapsulated Systems		Conjugated Systems					
	0.01 M PBS, pH 7.4		0.01 M PBS, pH 7.4			Vitreous/0.01 M PBS, pH 7.4 (50/50, *v*/*v*)		
	P[KF]_D,L_-2(DEX)	P[KF]_D,L_-2(DEX)/HEP	P[KF]_D,L_-2/DEX	P[KF]_D,L_-2/DEX/HEP	P[EF]-2/DEX	P[KF]_D,L_-2/DEX	P[KF]_D,L_-2/DEX/HEP	P[EF]-2/DEX
Zero-order *	R^2^ = 0.9644	R^2^ = 0.9565	R^2^ = 0.9653	R^2^ = 0.9985	R^2^ = 0.9517	R^2^ = 0.9811	R^2^ = 0.9937	R^2^ = 0.9616
	K_zo_ = 14.32	K_zo_ = 13.79	K_zo_ = 0.764	K_zo_ = 0.598	K_zo_ = 0.820	K_zo_ = 1.564	K_zo_ = 1.568	K_zo_ = 0.782
First-order *	R^2^ = 0.9950	R^2^ = 0.9924	R^2^ = 0.9945	R^2^ = 0.9962	R^2^ = 0.9907	R^2^ = 0.9943	R^2^ = 0.9956	R^2^ = 0.9888
	k_fo_ = 0.246	k_fo_ = 0.231	k_fo_ = 1.2 × 10^−2^	k_fo_ = 7.9 × 10^−3^	k_fo_ = 1.4 × 10^−2^	k_fo_ = 2.3 × 10^−2^	k_fo_ = 2.3 × 10^−2^	k_fo_ = 1.3 × 10^−2^
Higuchi *	R^2^ = 0.9896	R^2^ = 0.9805	R^2^ = 0.9941	R^2^ = 0.9675	R^2^ = 0.9896	R^2^ = 0.9892	R^2^ = 0.9801	R^2^ = 0.9932
	K_H_ = 29.629	K_H_ = 28.418	K_H_ = 6.085	K_H_ = 4.486	K_H_ = 6.558	K_H_ = 8.714	K_H_ = 8.562	K_H_ = 6.600
Korsmeyer-Peppas *	R^2^ = 0.9926	R^2^ = 0.9845	R^2^ = 0.9948	R^2^ = 0.9990	R^2^ = 0.9885	R^2^ = 0.9945	R^2^ = 0.9964	R^2^ = 0.9932
	K_KP_ = 25.842	K_KP_ = 23.627	K_KP_ = 3.480	K_KP_ = 0.752	K_KP_ = 3.936	K_KP_ = 5.422	K_KP_ = 3.393	K_KP_ = 4.375
	n = 0.605	n = 0.641	n = 0.643	n = 0.947	n = 0.631	n = 0.645	n = 0.780	n = 0.602
Hixon-Crowell *	R^2^ = 0.9900	R^2^ = 0.9858	R^2^ = 0.9884	R^2^ = 0.9981	R^2^ = 0.9826	R^2^ = 0.9920	R^2^ = 0.9965	R^2^ = 0.9827
	K_HC_ = 7.0 × 10^−2^	K_HC_ = 6.6 × 10^−2^	K_HC_ = 3.5 × 10^−3^	K_HC_ = 2.4 × 10^−3^	K_HC_ = 3.9 × 10^−3^	K_HC_ = 6.8 × 10^−3^	K_HC_ = 6.7 × 10^−3^	K_HC_ = 3.7 × 10^−3^
Hopfenberg *	R^2^ = 0.9950	R^2^ = 0.9924	R^2^ = 0.9945	R^2^ = 0.9990	R^2^ = 0.9907	R^2^ = 0.9943	R^2^ = 0.9960	R^2^ = 0.9888
	K_Hb_ = 1.8 × 10^−4^	K_Hb_ = 6.2 × 10^−5^	K_Hb_ = 3.4 × 10^−6^	K_Hb_ = 4.8 × 10^−3^	K_Hb_ = 5.9 × 10^−6^	K_Hb_ = 5.2 × 10^−5^	K_Hb_ = 2.3 × 10^−3^	K_Hb_ = 4.3 × 10^−6^
Baker-Lonsdale *	R^2^ = 0.9822	R^2^ = 0.9736	R^2^ = 0.9913	R^2^ = 0.9590	R^2^ = 0.9882	R^2^ = 0.9840	R^2^ = 0.9713	R^2^ = 0.9909
	K_BL_ = 2.0 × 10^−2^	K_BL_ = 1.8 × 10^−2^	K_BL_ = 7.6 × 10^−4^	K_BL_ = 3.8 × 10^−4^	K_BL_ = 9.1 × 10^−4^	K_BL_ = 1.6 × 10^−3^	K_BL_ = 1.5 × 10^−3^	K_BL_ = 9.5 × 10^−4^
Weibull **	R^2^ = 0.9963	R^2^ = 0.9940	R^2^ = 0.9987	R^2^ = 0.9992	R^2^ = 0.9990	R^2^ = 0.9963	R^2^ = 0.9987	R^2^ = 0.9912
	α = 2.51	α = 2.26	α = 53.228	α = 587.182	α = 23.440	α = 325.204	α = 191.949	α = 58.592
	β = 0.64	β = 0.51	β = 0.887	β = 1.345	β = 0.716	β = 1.504	β = 1.379	β = 0.943
Gompertz **	R^2^ = 0.9960	R^2^ = 0.9984	R^2^ = 0.9911	R^2^ = 0.9901	R^2^ = 0.9967	R^2^ = 0.9875	R^2^ = 0.9923	R^2^ = 0.9763
	α = 1.442	α = 1.529	α = 39.371	α = 168.455	α = 16.931	α = 213.930	α = 223.724	α = 30.684
	β = 1.903	β = 1.822	β = 2.390	β = 3.007	β = 1.954	β = 3.942	β = 3.935	β = 2.320
Peppas-Sahlin **	R^2^ = 0.9866	R^2^ = 0.9782	R^2^ = 0.9992	R^2^ = 0.9934	R^2^ = 0.9980	R^2^ = 0.9971	R^2^ = 0.9984	R^2^ = 0.9970
	K_1_ = 34.304	K_1_ = 32.556	K_1_ = 3.146	K_1_ = 18.141	K_1_ = 3.665	K_1_ = 3.697	K_1_ = 3.329	K_1_ = 4.107
	K_2_ = 2.965	K_2_ = 2.778	K_2_ = 0.025	K_2_ = 14.802	K_2_ = 0.037	K_2_ = 0.032	K_2_ = 0.026	K_2_ = 0.018
	m = 0.492	m = 0.484	m = 0.707	m = 0.210	m = 0.696	m = 0.821	m = 0.835	m = 0.631

* 60% of release; ** full curve modelling.

## Data Availability

The data are available within the article or its Supplementary Materials.

## Supplementary Materials

The supporting information can be downloaded at: https://www.mdpi.com/article/10.3390/ijms24043702/s1.
