# Supplementary material for: Synthesis and Characterization of Nanoparticle-Based Dexamethasone-Polypeptide Conjugates as Potential Intravitreal Delivery Systems
Source: Int J Mol Sci. 2023 Feb 12;24(4):3702. doi: 10.3390/ijms24043702 (PMC9962198; doi:10.3390/ijms24043702)
Supplement: Supplementary file 1 [file ijms-24-03702-s001.zip › ijms-2116495-supplementary.pdf]

Supplementary Materials

Synthesis and Characterization of Nanoparticle-Based Dexamethasone-Polypeptide Conjugates as Potential Intravitreal Delivery Systems

Natalia Zashikhina [1], Sergei Gladnev [2], Vladimir Sharoyko [2,3], Viktor Korzhikov-Vlakh [2], Evgenia Korzhikova-Vlakh [1,2,*], Tatiana Tennikova [2]

1. Institute of Macromolecular Compounds, Russian Academy of Sciences, St. Petersburg 199004, Russia
2. Institute of Chemistry, Saint-Petersburg State University, St. Petersburg 198504, Russia
3. Department of General and Bioorganic Chemistry, Pavlov First Saint-Petersburg State Medical University, St. Petersburg 197022, Russia

* Correspondence: vlakh@hq.macro.ru (E.K.-V.)

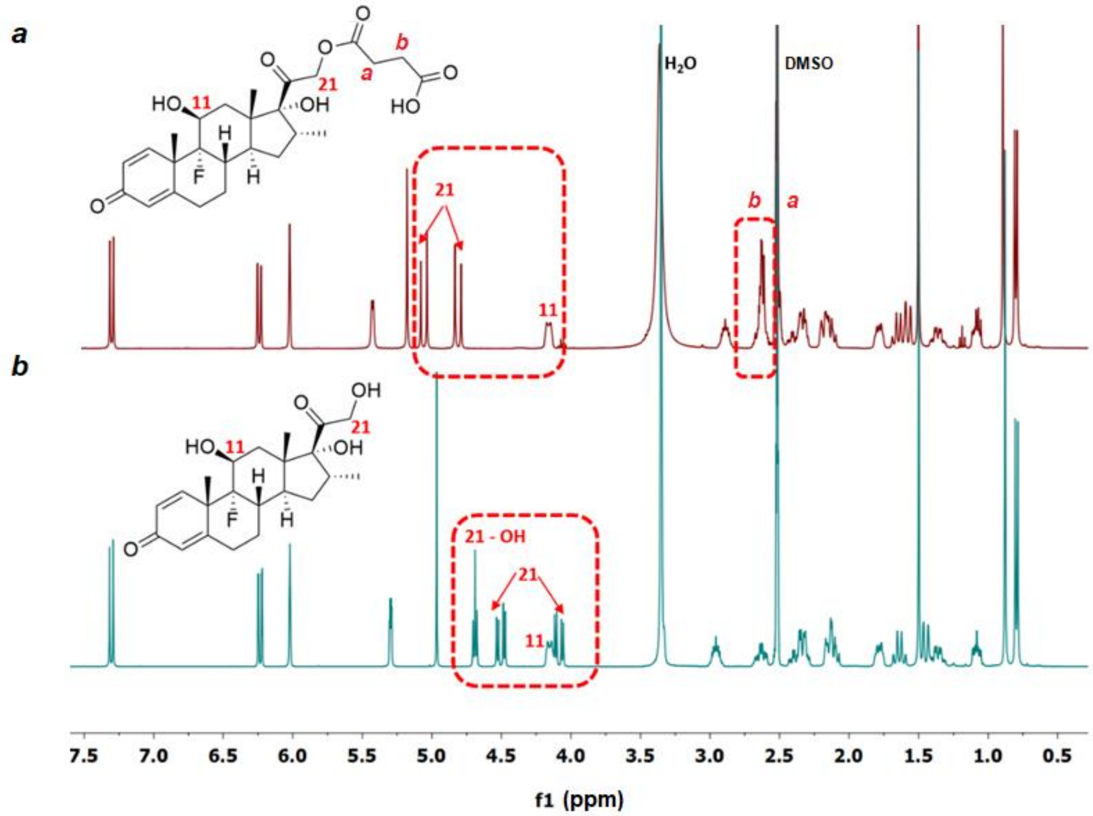

Figure S1. ^{1}H NMR spectra of DEX-hemisuccinate **(a)** and DEX **(b)** (DMSO-d_6).

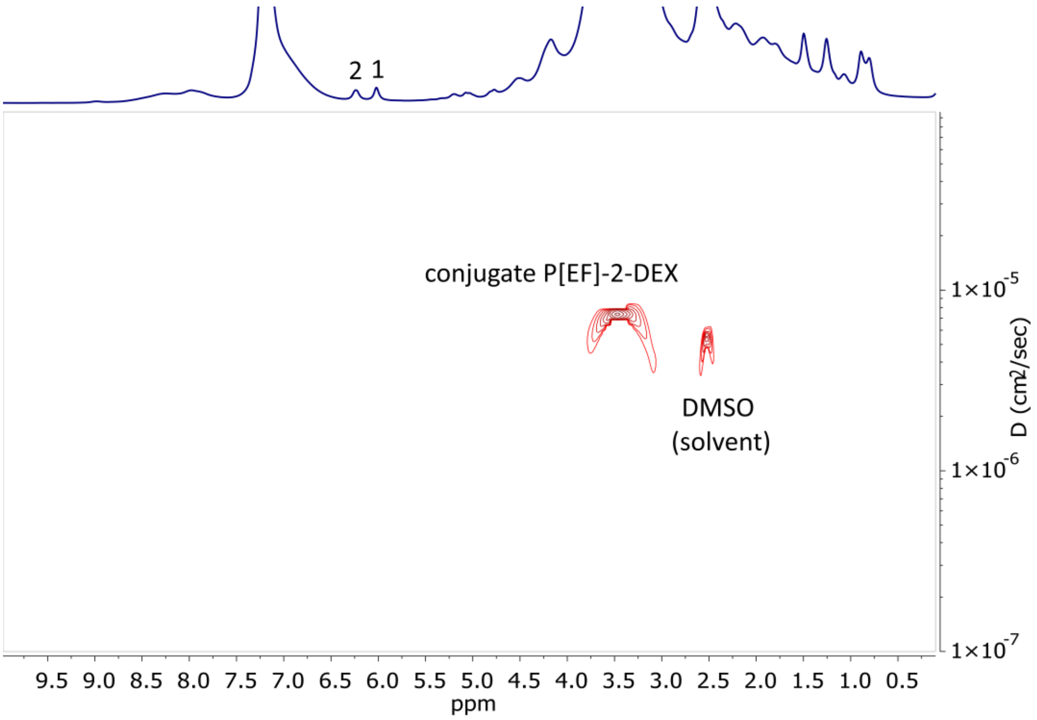

Figure S2. ^{1}H NMR DOSY spectrum of P[EF]-2-DEX. In spectrum 1,2 - hydrogen atoms at 4 and 2 carbon atoms of DEX, respectively (DMSO-d6).

(a)

Zero-order	$F=k_0*t$
First-order	$F=100*[1-Exp(-k_1*t)]$
Higuchi	$F=k_H*t$^0.5
Korsmeyer-Peppas	$F=k_{KP}*t$^n
Hixson-Crowell	$F=100*[1-(1-k_{HC}*t)$^3]
Hopfenberg	$F=100*[1-(1-k_{HB}*t)$^n]
Baker-Lonsdale	3/2*[1-(1-F/100)^(2/3)]-F/100=$k_{BL}*t$
Peppas-Sahlin	F=k1*t^m+k2*t^(2*m)
Weibull	F=100*{1-Exp[-((t-Ti)^β)/α]}
Gompertz	F=100*Exp{-α*Exp[-β*log(t)]}

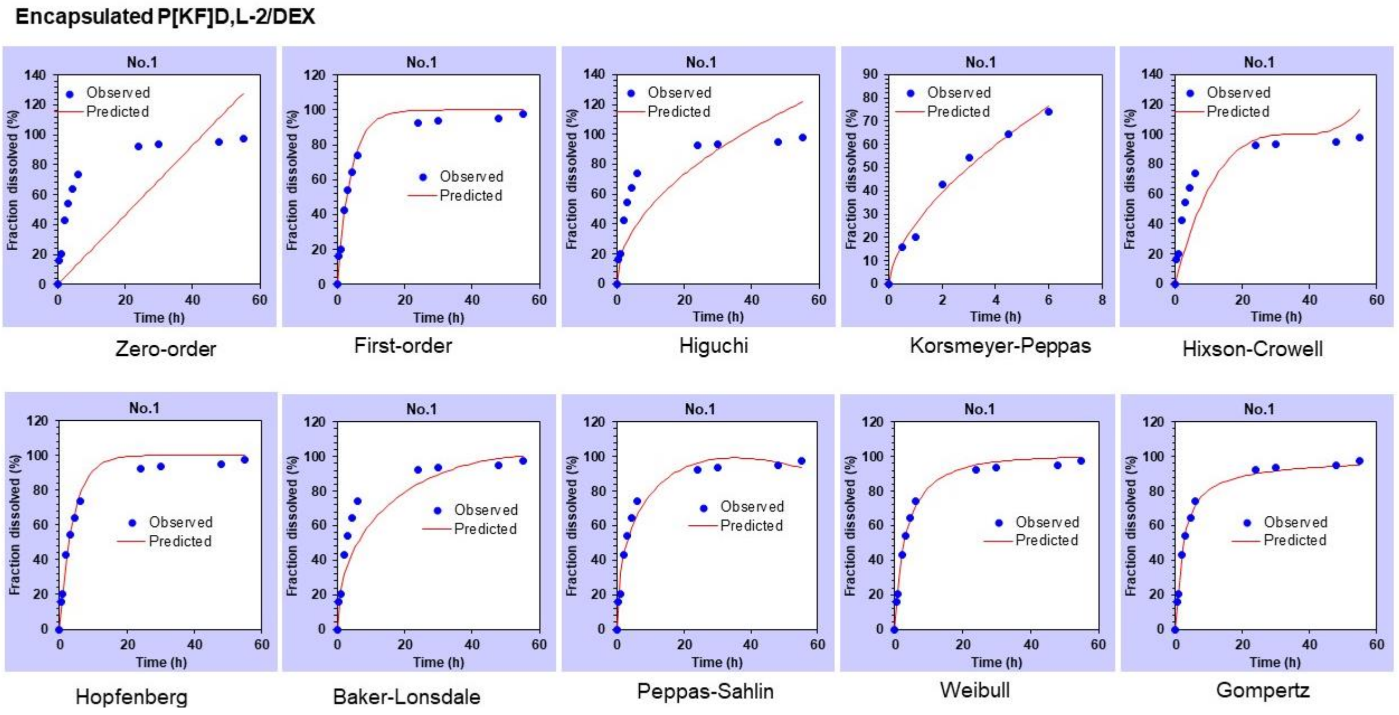
Encapsulated P[KF]D,L-2/DEX
No.1
Observed
Predicted
Fraction dissolved (%)
Time (h)
Zero-order
First-order
Higuchi
Korsmeyer-Peppas
Hixson-Crowell
Hopfenberg
Baker-Lonsdale
Peppas-Sahlin
Weibull
Gompertz

(b)

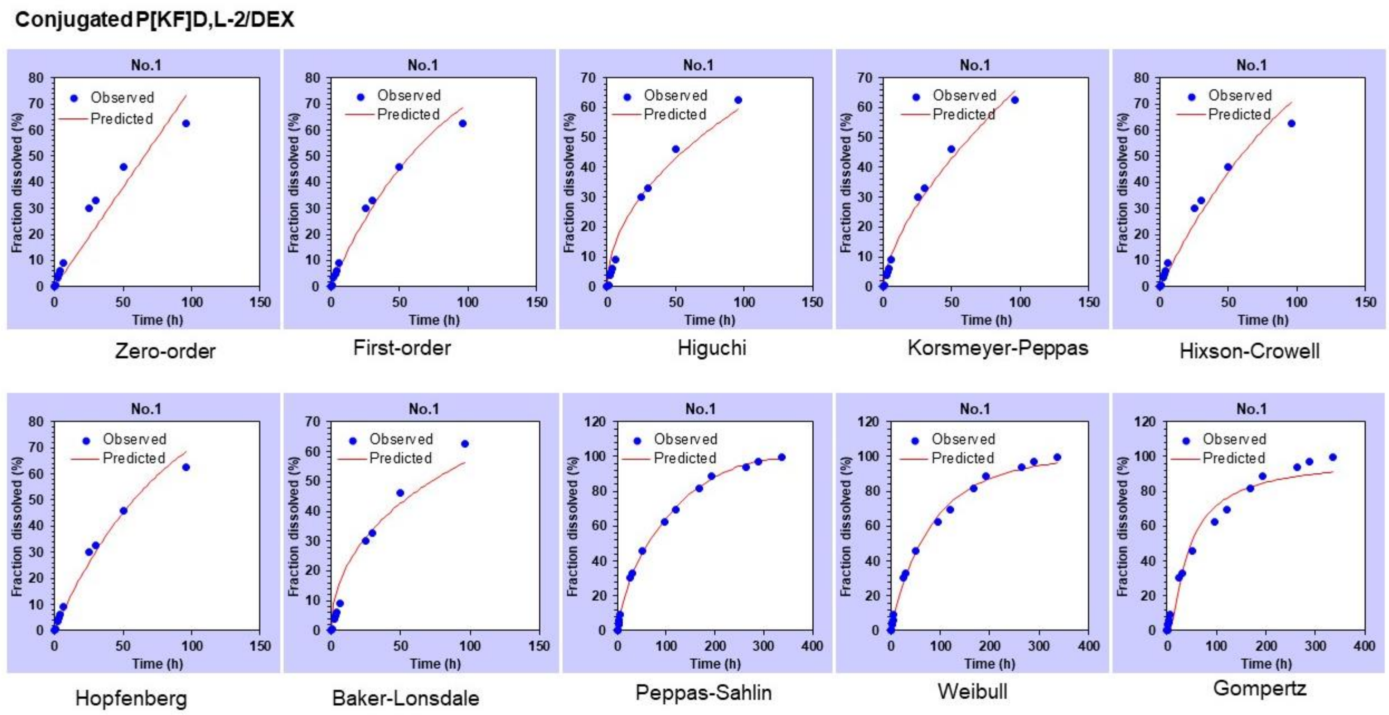
Conjugated P[KF]D,L-2/DEX
No.1
Observed
Predicted
Fraction dissolved (%)
Time (h)
Zero-order
First-order
Higuchi
Korsmeyer-Peppas
Hixson-Crowell
Hopfenberg
Baker-Lonsdale
Peppas-Sahlin
Weibull
Gompertz

(c)

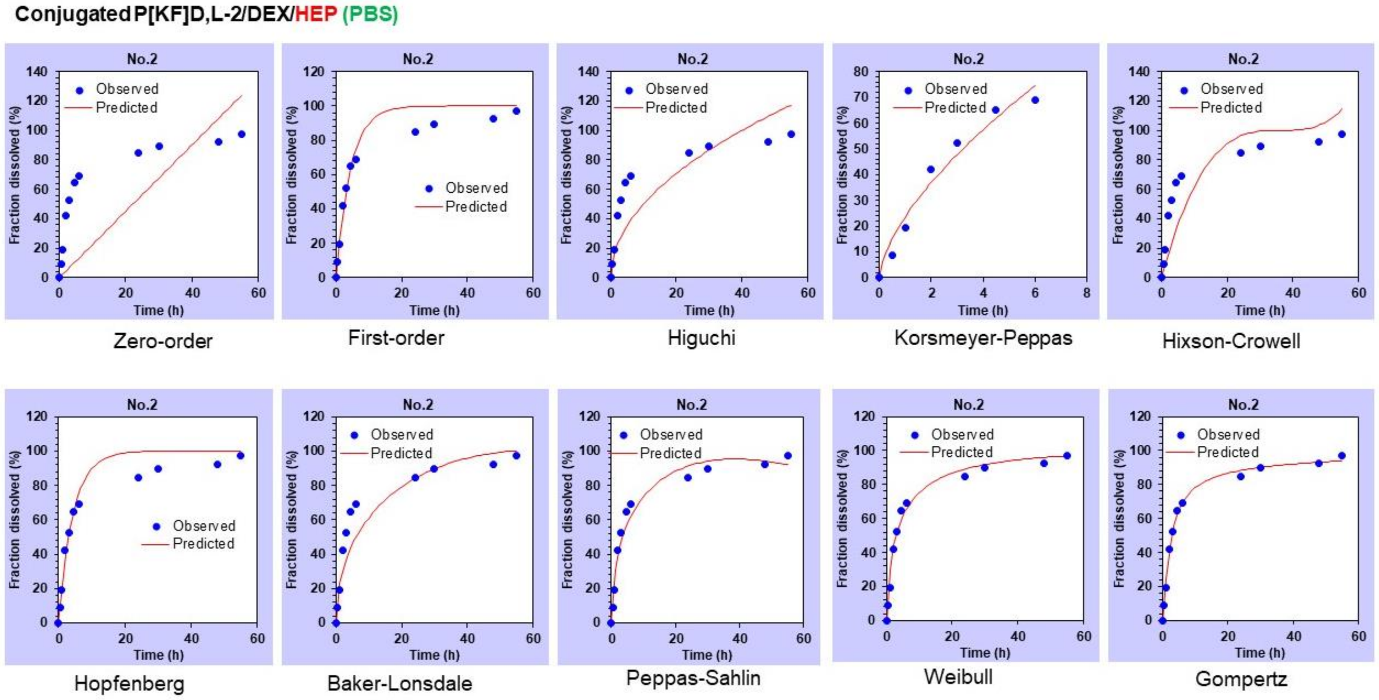

(d)

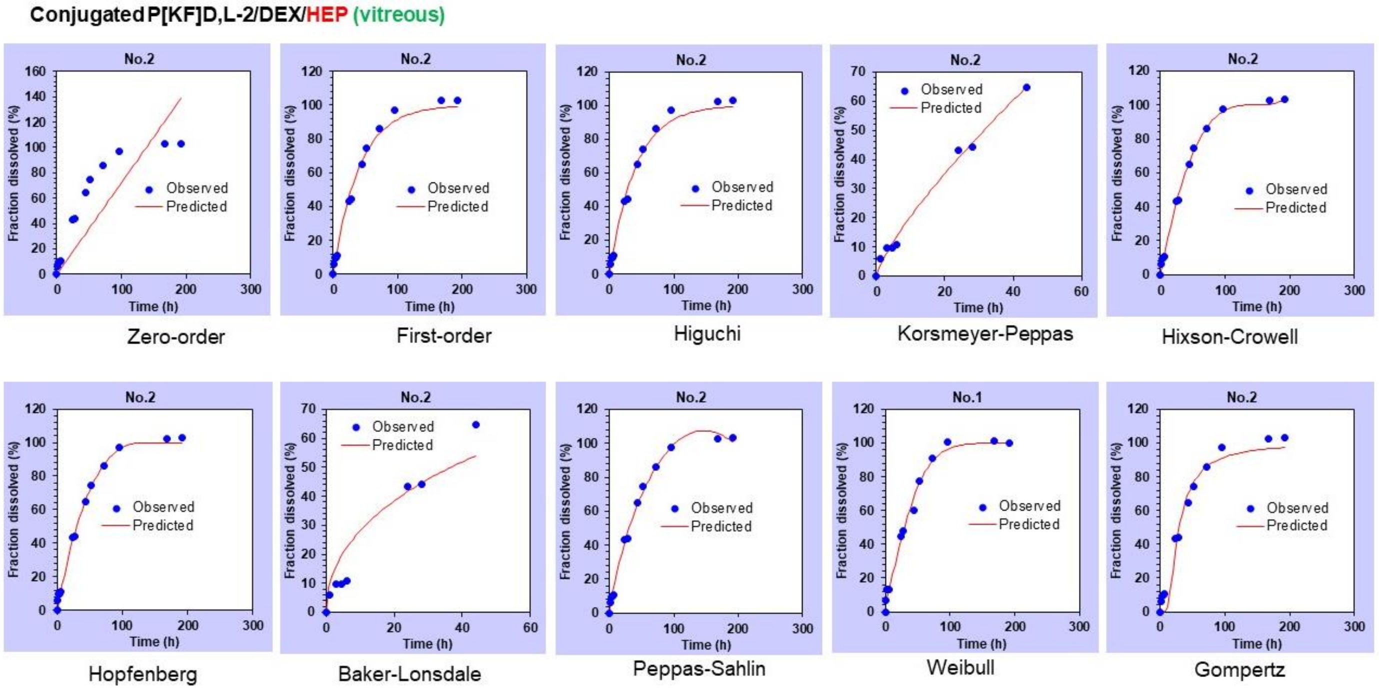

(e)

Figure S3 Equations for the different release models **(a)** and mathematical analysis for the release data **(b-e)** for some encapsulated and conjugated samples.

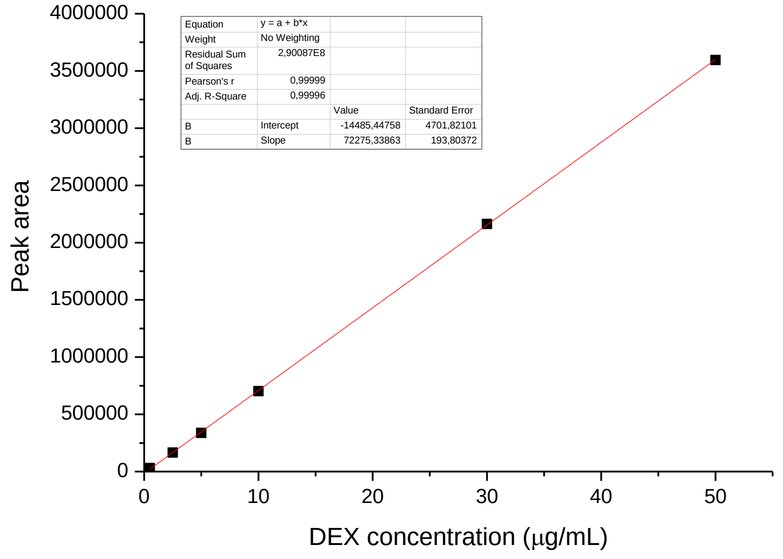

Figure S4. HPLC calibration plot obtained by reversed-phase HPLC at isocratic elution (C18 column, acetonitrile/water = 30/70, *v*/*v*) for dexamethasone solutions with different concentrations.

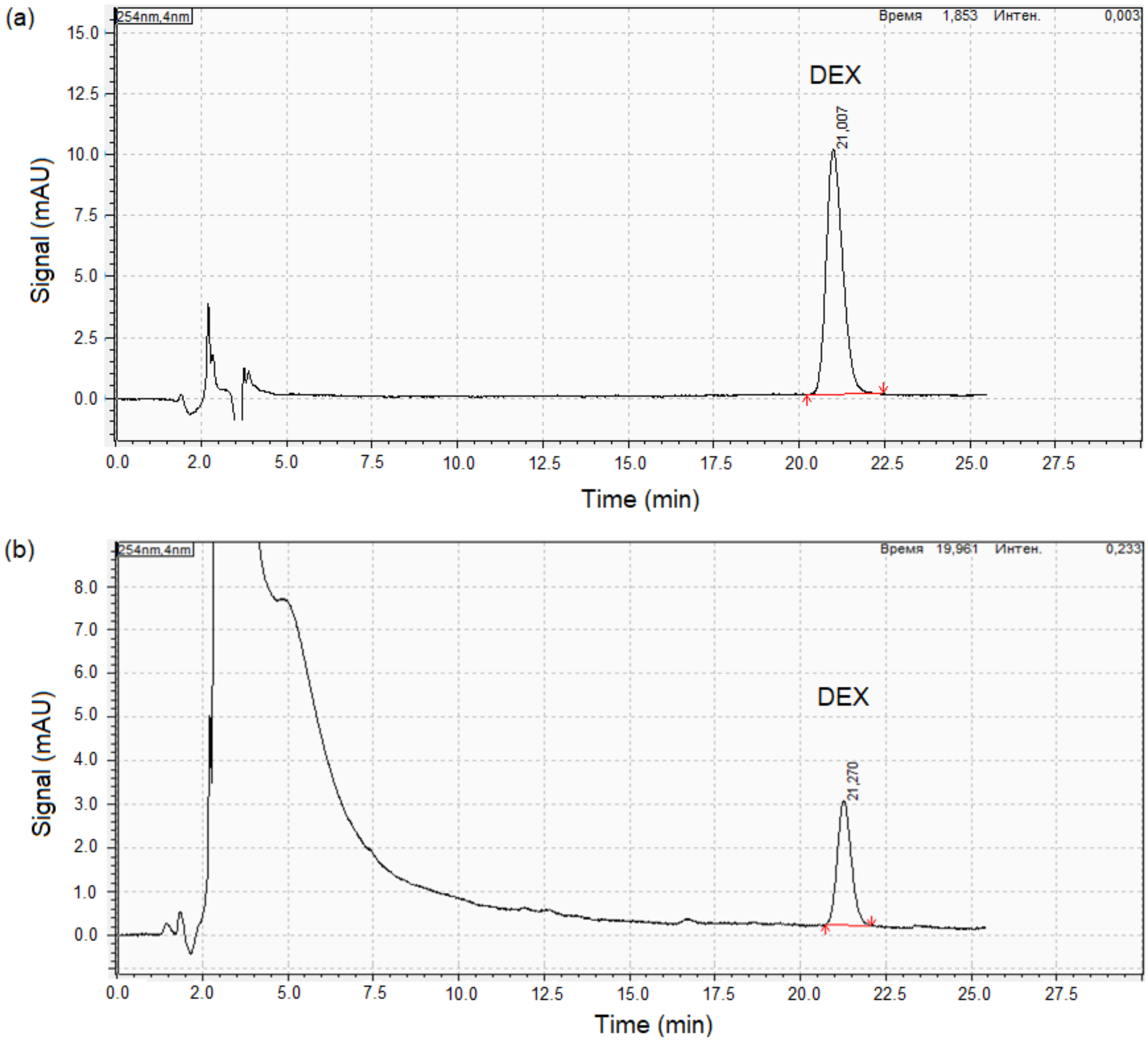

Figure S5. Chromatograms for standard DEX solution **(a)** and solution obtained after DEX release **(b)**.
